# Exploring New and Potential Indications for Botulinum Toxin Treatment: An Updated Literature Review

**DOI:** 10.7759/cureus.75549

**Published:** 2024-12-11

**Authors:** Jimmy Wen, Dawnica Nadora, Ubaid Ansari, Burhaan Syed, Mouhamad Shehabat, Daniel I Razick, Adam A Razick, Thiru Rajagopal

**Affiliations:** 1 Physical Medicine and Rehabilitation, California Northstate University College of Medicine, Elk Grove, USA; 2 Dermatology, California Northstate University College of Medicine, Elk Grove, USA; 3 Neurology, California Northstate University College of Medicine, Elk Grove, USA; 4 Surgery, California Northstate University College of Medicine, Elk Grove, USA; 5 Life Sciences, University of California, Los Angeles, Los Angeles, USA; 6 General Surgery, Mercy General Hospital, Sacramento, USA

**Keywords:** botox injections, botulinum (botox), botulinum neurotoxin type-a, botulinum toxin, connective tissue disease

## Abstract

Botulinum toxin (BoNT) has traditionally been utilized to relieve tension in muscular and connective tissue diseases (CTD). However, its usage has rapidly expanded and now encompasses usage for neurological, gastrointestinal, psychological, cardiovascular, ophthalmology, orthopedics, and more. More recently, its usage has been utilized for sequelae of CTDs such as Raynaud's disease and reduced oral aperture secondary to scleroderma/systemic sclerosis. Beyond its current applications, BoNT holds promise in various medical fields but is not FDA-approved in these conditions. Thus, the design and conduction of well-designed randomized controlled trials are essential in establishing the efficacy and safety of BoNT treatment which can help accelerate regulatory approval for new indications. The versatility of BoNT suggests that its therapeutic applications continue to expand, offering novel therapies for a wide range of conditions. This review aims to comprehensively evaluate the literature on BoNT’s current FDA-approved indications and potential non-FDA uses in other medical conditions. Additionally, this review offers potential insights and future possibilities for BoNT treatment.

## Introduction and background

Botulinum toxin (BoNT), a protein neurotoxin produced by *Clostridium botulinum* is an acetylcholine release inhibitor that induces a neuromuscular blocking effect initially used for muscular conditions with increased tension [1]. BoNT relieves increased tension in muscular and connective tissue diseases by stimulating temporary denervation [1]. Its effects typically peak around one to two weeks, with neuronal activity returning around three months after injection [1]. Previous studies have also shown that BoNT can produce sustained flaccid paralysis of striated muscle for two to six months [2]. However, its usage has now expanded to many more indications such as in dermatology, urology, gastrointestinal, ophthalmology, orthopedics, and more [1,3]. Over the last two decades, BoNT has become a promising alternative therapy as a nonsurgical treatment for vasospastic disease. Its mechanism has been linked to decreased conversion of fibroblasts to myofibroblasts and increased matrix metalloproteinase [4]. Other proposed mechanisms include sympathetic adrenergic and cholinergic vasoconstriction inhibition and endothelial exocytosis of endothelin-1 [5]. BoNT decreases the expression of connective tissue growth factor (CTGF) and transforming growth factor-b1 (TGF-b1), which inhibits cellular adhesion, growth, collagen deposition, extracellular matrix production, and fibroblast growth [2,6].

Its mechanism of action of inducing paralysis has led to its possible therapeutic usage in connective tissue diseases (CTD) such as systemic sclerosis (SSc) and Raynaud’s disease (RD) to improve quality of life and functionality [4]. Symptom control includes combination therapy with physical therapy and pharmacological agents such as calcium channel blockers, prostacyclin analogs, phosphodiesterase inhibitors, and endothelin receptor antagonists. However, limited options are available if treatment fails or the patient cannot tolerate adverse effects. Surgical treatment is time and skill-intensive and is generally not curative [4,5]. In addition to CTDs, BoNT has been injected into the upper esophageal sphincter with success for dysphagia in patients with inclusion body myositis [7]. Thus, BoNT can be a useful intervention for patients who do not currently require surgery or those who cannot undergo surgery due to comorbidities that increase the risk of surgical/anesthesia complications. 

Since the inception of BoNT, its usage and applications in various fields have continued to grow. This review aims to comprehensively evaluate the literature on BoNT’s current indications and potential uses in other muscular/connective tissue disorders, CTDs, and their associated sequelae. Additionally, this review offers potential insights and future possibilities for BoNT treatment. 

## Review

BoNT

BoNT interferes with nerve function by inhibiting the release of acetylcholine from the presynaptic neuron, resulting in flaccid paralysis. Acetylcholine modulates muscle contraction at the neuromuscular junction, which is the connection point between a motor neuron and a muscle fiber [8,9]. There are several forms of botulism each associated with different routes of toxin exposure including foodborne botulism, wound botulism, and infant botulism. Foodborne botulism typically results from ingesting improperly preserved or canned foods, where the bacteria produce the toxin in an anaerobic environment [10]. Wound botulism occurs when *C. botulinum* spores infect an open wound and can be seen especially in patients who inject drugs [11]. Infant botulism arises from the ingestion of spores in foods such as honey that germinate and produce toxins in the immature gut [12]. Symptoms of botulism include muscle weakness, difficulty breathing, and sometimes death due to respiratory failure [10]. Treatment involves administering antitoxin and providing supportive care such as mechanical ventilation to manage the effects of paralysis until nerve function recovers. Early diagnosis is critical for preventing severe outcomes.

Safety profile of BoNT

First approved by the United States Food and Drug Administration (FDA) in 1989, BoNT has become a mainstay in medicine for various diseases and conditions [13]. BoNT specifically types A and B, is clinically used for various medical and cosmetic purposes. BoNT-A is produced by *C. botulinum*, consisting of a heavy chain (100 kDa) and a light chain (50 kDa) connected by a disulfide bond [14]. Among the seven serotypes of BoNT, only BoNT-A and BoNT-B are approved for clinical use, with BoNT-A being the most potent. The mechanism of BoNT-A involves four fundamental stages: binding to cholinergic neuron receptors, internalization via receptor-mediated endocytosis, disulfide reduction and translocation of the light chain, and inhibition of acetylcholine release [14]. BoNT-A specifically works by blocking the SNARE protein SNAP-25, preventing neuromuscular transmission and causing muscle paralysis. The internalization process is optimal after about 90 minutes at room temperature. BoNT-A is particularly effective in dermatology for reducing facial wrinkles, lifting eyebrows, and treating conditions like hyperhidrosis, lichen simplex, dyshidrotic eczema, and acne [15]. The toxin works by blocking acetylcholine release, leading to temporary muscle paralysis lasting three to six months, with an optimal cosmetic dose of 20 units. BoNT is considered safe and has minimal adverse effects, although its impact gradually diminishes over time. 

With a known medial lethal dose (LD50) of 1 nanogram of toxin per kilogram body mass, BoNT is the most lethal substance on a weight basis [10]. However, current literature states that the median lethal dose of BoNT used at present is primarily an estimate of human safety and does not reflect the potential for acute or chronic toxicity, such as effects on the brain or other organs, which may occur at lower doses or with prolonged administration [16]. Generally, underdosing is recommended since the dose-dependent side effects of BoNT injection vary in the pharmacokinetic effects of different organs. In addition, administering multiple injections at lower doses can minimize the risk of systemic side effects, such as difficulty with drinking and talking, nausea, vomiting, and diarrhea, while still achieving the desired outcomes [16]. If needed, naturally occurring botulinum intoxication in adults and children can be treated with antitoxin therapy if the intervention is administered early in the course of the illness. As of 2013, the only antitoxin available is the q Heptavalent Botulinum Antitoxin (HBAT), developed by the Centers for Disease Control and Prevention (CDC) and Emergent BioSolutions Inc. (Gaithersburg, Maryland, United States), which targets all seven known serotypes of BoNT (A through G) [16].

FDA-approved indications

BoNT has been utilized by clinicians since the late 1970s [8,13]. While there are FDA-approved indications for BoNT use, it is also commonly used off-label for other conditions, which will be discussed later in this review. Several indications will be discussed further.

Strabismus/Blepharospasm

Alan Scott first used BoNT to treat strabismus in 1977 and it is now indicated to treat patients 12 years old and older with strabismus and blepharospasm caused by dystonia [17]. Strabismus is an eye movement and alignment disorder due to muscle imbalances that cause the eyes to be in different directions [18]. Although a recognized treatment, it is no longer the primary treatment for this condition since repeated injections are necessary to be effective [19]. Surgery is now the favored approach, though BoNT may be used temporarily or when surgery is not preferred. However, if necessary, its long-term treatment is safe and effective [20]. 

Chronic Migraines 

In 2010, the FDA approved using BoNT for chronic migraines [21]. Randomized, double-blind, placebo-controlled trials have shown that BoNT effectively treats chronic migraines [22]. In evaluating the safety and efficacy of BoNT-A as headache prophylaxis in adults with chronic migraine, Aurora et al. reported a statistically significant reduction in headache-day frequency compared to placebo in two phase III clinical trials involving 1,384 patients [23]. BoNT is currently indicated for adult patients experiencing migraines occurring 15 days or more per month lasting four hours or longer each each day [22]. However, its use for episodic migraines or in children has not been established. 

Wrinkles and Axillary Hyperhidrosis 

In aesthetic medicine, BoNT was first approved in Canada in 2000 to treat focal muscle spasticity and for cosmetic wrinkle reduction [24]. The FDA approved BoNT for cosmetic injections in 2002 and was limited to glabellar lines, lateral canthal lines, and forehead lines [25]. Injecting BoNT into the underlying muscles causes them to relax, thereby reducing the overlying lines on the skin [25]. In addition, BoNT is also indicated hyperhidrosis, a condition characterized by excessive sweating in areas such as the axillae, palms, and soles. When BoNT is injected into exocrine gland tissues, it reduces the production of sweat, saliva, and tears, thus it is used when topical aluminum chlorides or systemic anticholinergic treatments, such as oxybutynin and glycopyrrolate, prove ineffective [21,26]. 

Sialorrhea

Most recently, BoNT was indicated for sialorrhea treatment in 2018 for adults and in 2020 for children over two years of age [27]. BoNT injections are typically done transcutaneously into the salivary glands where it blocks the release of acetylcholine presynaptically in the parasympathetic ganglia, thereby reducing saliva secretion for about three to four months [28]. In a 2011 comparative trial between BoNT-A and BoNT-B, it was reported that both were equally effective in treating sialorrhea in patients with neurological conditions, specifically amyotrophic lateral sclerosis (ALS) and Parkinson’s disease (PD) [29]. However, BoNT-A demonstrated a safer profile than BoNT-B, with fewer adverse effects observed during treatment. Nevertheless, a multicenter, randomized, double-blind, placebo-controlled study with 54 PD subjects experiencing sialorrhea showed that intraglandular injection of BoNT-B was safe, tolerable, and efficacious in treating sialorrhea in PD patients compared to placebo treatment [30]. 

Spasticity, Cervical Dystonia

BoNT is also indicated for the treatment of spasticity and cervical dystonia. Spasticy can be caused by conditions such as multiple sclerosis, cerebral palsy, and brain or spinal cord trauma, leading to an abnormal increase in muscle contraction and muscle ton [31]. BoNT injections were approved by the FDA in 2010 and were subsequently approved for limb spasticity in 2015 [31]. Santameto et al. conducted several studies to determine the efficacy of high-dose injections in stroke patients to reduce upper and lower limb spasticity. A 2013 study first explored the use of high BoNT doses (up to 840 units), revealing notable significant decreases in spasticity and pain and improved disability outcomes, without reports of adverse events [32]. The same research team later confirmed the efficacy and safety of these high doses in a 2016 study, and in 2017, they expanded their findings, showing the long-term safety and sustained effectiveness of repeated high-dose treatments over two years [33-34].

Cervical dystonia (CD), a neurological disorder characterized by involuntary muscle contractions in the neck and shoulders, has been treated with BoNT since 2000. It inhibits acetylcholine release at neuromuscular junctions, causing muscle weakness in the targeted muscles [35]. A 2020 Cochrane Review of randomized, placebo-control trials for BonT treatment for cervical dystonia has shown that a single treatment is effective in improving cervical dystonia symptoms [36]. Interestingly, a 2024 phase 3, randomized, double-blind, placebo-controlled study to assess the efficacy, duration of response, and safety of DaxibotulinumtoxinA for Injection (DAXI), a new BoNT-A formulation, in subjects with CD. This study reported that DAXI, administered at 125U and 250U, is an effective, safe, long-acting, and well-tolerated treatment for CD [37].

Detrusor Overactivity

In 2012, the FDA approved the use of BoNT for neurogenic detrusor overactivity (NDO), which typically leads to conditions such as urinary incontinence (UI), overactive bladder syndrome, and non-neurogenic overactive bladder syndrome [25]. In a 24-week study randomized control trial, it was demonstrated that intramuscular injections of BoNT-A into the detrusor can provide effective, well-tolerated, clinically significant reductions in the signs and symptoms of urinary incontinence caused by neurogenic detrusor overactivity [38]. In a follow-up study by Schurch et al., they reported that BoNT can be used to lower urinary incontinence frequency in adults and children with urinary incontinence due to NDO [39]. The currently FDA-approved indications can be found in Table 1.

**Table 1:** FDA-approved indications for botulinum toxin †Dosing varies based on location, severity, prior response, and adverse event history

Systems	Conditions	Botulinum Dosage	Trade/Generic Name
Neurological Conditions	Blepharospasm [17]	1.25 Units - 2.5 Units into 3 sites per affected eye	Botox (OnabotulinumtoxinA)
			Xeomin (IncobotulinumtoxinA)
	Chronic Migraine [21-23]	155 Units across 31 injection sites	Botox (OnabotulinumtoxinA)
	Cervical Dystonia† [31-34]	200-300 Units across affected muscles	Botox (OnabotulinumtoxinA)
			Xeomin (IncobotulinumtoxinA)
			Dysport (AbobutlinumtoxinA)
			Myobloc (RimabotulinumtoxinB)
	Upper/Lower Limb Spasticity† [35-37]	Up to 400 Units across affected muscles	Botox (OnabotulinumtoxinA)
			Xeomin (IncobotulinumtoxinA)
			Dysport (AbobutlinumtoxinA)
			Myobloc (RimabotulinumtoxinB)
Ophthalmological Conditions	Strabismus [17-20]	Strabismus†: 1.25 - 2.5 Units in affected muscles	Botox (OnabotulinumtoxinA)
Dermatological Conditions	Glabellar Lines [24-26]	20 Units across 5 injection sites	Botox (OnabotulinumtoxinA)
			Xeomin (IncobotulinumtoxinA)
			Dysport (AbobutlinumtoxinA)
			Jeuveau (PrabotulinumtoxinA-xvfs)
	Lateral Canthal Lines [24-26]	24 Units across 3 injection sites on each side	Botox (OnabotulinumtoxinA)
	Forehead Lines [24-26]	20 Units across 5 injection sites	Botox (OnabotulinumtoxinA)
	Axillary Hyperhidrosis [24-26]	50 Units per axilla	Botox (OnabotulinumtoxinA)
			Dysport (AbobutlinumtoxinA)
Other Conditions	Sialorrhea [27-30]	100 Units across 4 injection sites	Botox (OnabotulinumtoxinA)
			Xeomin (IncobotulinumtoxinA)
Urological Conditions	Detrusor Overactivity [25,38]	200 Units across 30 sites in detrusor	Botox (OnabotulinumtoxinA)
			Jeuveau (PrabotulinumtoxinA-xvfs)
	Overactive Bladder [39]	100 Units across 20 sites in detrusor	Botox (OnabotulinumtoxinA)

Non-FDA approved uses

Musculoskeletal

Since its inception, BoNT has been used in therapeutic and cosmetic treatments for muscular and connective tissue disorders. The toxin’s ability to block conduction to muscles is useful in certain muscle dysfunctions. For example, in the setting of cervical dystonia, continuous involuntary contraction of neck muscles can occur, leading to either repetitive movements or irregular neck and head posturing, which can be detrimental to the patient’s standard of living. Various studies have assessed the safety and efficacy of BoNT-A, and the toxin was approved for usage in cervical dystonia in 2000 [24]. In a study done by Mohammadi et al., long-term BoNT-A treatment remained efficacious and safe over 14 years [40]. In patients who developed immunological resistance to BoNT-A, switching to BoNT-B often restored efficacy. One study showed that BoNT-B given at the same dose as BoNT-A provided similar results in terms of efficacy [41]. However, other studies showed greater concentrations of BoNT-B were required to exhibit the same effect. Additionally, there was a higher frequency of dry mouth and injection pan, with these effects lasting longer than BoNT-A [42]. 

Other muscular conditions that use BoNT therapeutically include spasticity, bruxism, and temporomandibular joint (TMJ) disorder. In a study done by Chen et al., BoNT-A was used in focal injections to the biceps brachii muscle in patients who suffered a stroke and were left with spastic hemiplegia [43]. Spasticity assessment, through the Modified Ashworth Scale (MAS), reflex torque, and muscle and motor performance, were assessed over four weeks. After administration of the toxin, they found that spasticity and muscle strength were decreased in the spastic side, and unchanged in the contralateral arm. Motor performance remained unaffected by BoNT administration on either side. Various studies showed that injection of BoNT-A provided relief of bruxism symptoms, and patient-reported pain scores significantly improved after administration [44-46]. Similar findings were seen with TMJ disorders, providing relief of jaw pain and mandibular movement dysfunction. A study by Blanco-Rueda et al. showed that BoNT-A reduced pain, joint clicking, and headaches [47]. 

BoNT also has implications for wound healing, scarring, and cleft lip repair. To minimize scarring after a wound, an important factor is the tension from the skin acting on the edges of the wound during the healing process. Wound healing follows phases or steps that overlap, starting with homeostasis, then inflammatory and proliferative stages, and finally remodeling. Excessive tension can prolong the inflammatory portion of the system [48]. On a microscopic level, the tension exhibited by surrounding muscles and skin can result in the disruption of fibroblast cytoplasmic extensions, delaying healing [1]. This prolongation can also lead to increased collagen and glycosaminoglycans within the wound, leading to undesirable scar hypertrophy and hyperpigmentation [49]. A study done by Patil et al. showed a significant increase in patient-reported visual analog scale using BoNT-A compared to placebo after wound closure [50]. Treatment of hypertrophic scars with either BoNT-A or B has been studied as well, showing improvement in itching sensation and erythema [51]. BoNT’s function in inhibiting neurotransmitters, like glutamate and substance P, helped decrease downstream inflammatory effects as well, lessening scar hypertrophy [52]. Finally, BoNT can be used in cleft lip repair, due to increased upper lip tension following repair. As mentioned, BoNT's function in releasing tension can lead to favorable cosmetic and therapeutic effects on the closing of the wound. A study by Navarro-Barquin et al. used the toxin for infantile cleft lip repair and showed it to be safe and effective, along with showing acceptable aesthetic results with no complications [53]. 

Though its pathogenesis is presently unknown, SSc is an autoimmune CTD characterized by the overproduction of connective tissue components in the body. Often, this is an excess production of collagen, the primary fibrous protein used in connective tissues around the body [54]. In addition to the aforementioned complications such as pulmonary arterial hypertension, cardiomyopathy, scleroderma, and reduced oral aperture (ROA), patients may develop RD, neuropathic symptoms such as paresthesias and dysesthesias, digital ulcers, as well as contractures of skin tissue [54]. However, BoNT has been shown to provide short-term relief to patients suffering from SSc-related ROA in a small clinical trial of 17 females with SSc and ROA [4]. After being treated with BoNT-A, patients saw a substantial increase in oral aperture two weeks after the treatment was administered (p > 0.001) as well as reported improvements in quality of life. Of note, though, there were no further marked increases in oral aperture three months after the beginning of the trial. As such, while BoNT offers a temporary respite, it was not shown to be a long-term solution. 

A more heavily studied area related to the efficacy of BoNT is its treatment potential for CTD-associated RD. RD is a disorder that presents in patients as discolored fingers along with pain, swelling, and numbness after being exposed to low temperatures or stress. In severe cases, RD may even require amputation as a result of gangrene or infection [55]. As such, RD can have an extremely debilitating impact on patients’ quality of life. Studies have provided contrasting pieces of evidence for BoNT's effectiveness as a treatment option for RD. For example, one clinical trial that included patients with limited and diffuse scleroderma found a reduction in blood flow to the affected areas in patients with RD one month after being administered BoNT-A [5]. Similarly, a three-year retrospective study by Medina et al. evaluated patients with severe RD who responded positively to BoNT-A treatment, with a statistically significant reduction in pain in more than half of the patients in the study after one month and the number of weekly episodes of RD without any reported serious adverse events [3]. Notably, at the three-month follow-up, five out of the seven patients with basal ulcers had resolution of symptoms. A larger study with 29 patients with scleroderma found that at the final follow-up in four months, there was a significantly greater decrease in pain severity, paraesthesia, Raynaud’s condition score, number of ulcers, ulcer diameter, and increase in upper extremity function compared to placebo (all p<0.05) [56]. Additionally, a comprehensive 2023 literature review of 42 studies found considerable positive results (96.2% of patients) when patients with Raynaud’s phenomenon were treated with BoNT. Complications were minor and self-resolving, with the most common being transient hand weakness and injection site pain [57]. 

Plantar fasciitis is typically caused by overuse injury secondary to micro-tears of the plantar fascia caused by repetitive strain or from trauma and multifactorial causes as well [58]. Ahmad et al. examined BoNT’s effects on plantar fasciitis and found a significant function (Foot and Ankle Ability Measures) and pain (Visual Analog Scale) in the treatment group compared to placebo (p<0.05) [59]. At six months, for BoNT and placebo group, the mean Foot and Ankle Ability Measures score (36.3-73.8 versus 35.9-40.9) and pain scores (7.2-3.6 versus 8.4-7.9) were significantly better than placebo (p=0.01). Interestingly, there was also a lessened need for surgical treatment of plantar fasciitis compared to placebo (p<0.005) [59]. However, despite promising results in the literature for plantar fasciitis treatment, there are some varying results. Radovic suggested that this may be due to a misclassification of all plantar heel pain as plantar fasciitis rather than Plantar Heel Pain Syndrome [60]. In a study by Radovic, four distinct manifestations (tarsal tunnel syndrome, medial/lateral plantar nerve entrapment, Baxter’s neuritis, plantar fasciosis) reported in four patients appeared to demonstrate prolonged improvement of function and pain reduction via BoNT injection into the origin of the abductor hallucis and quadratus plantae [60]. A 2023 meta-analysis evaluating BoNT’s efficacy in treating fasciitis pain, with a significant mean difference (MD) in the visual analog scale of -2.59 (95% CI: -3.36,-1.82) [61]. Subgroup analysis also found efficacy in treating plantar fasciitis (MD = -3.34, 95% CI:-4.08, -2.78), lumbar back fasciitis (MD = -2.17, 95% CI: -3.82, -0.52), neck and shoulder fasciitis (MD = -1.49, 95% CI: -2.76, -0.22) with p-values of <0.001, 0.001, and 0.02, respectively [61]. BoNT displays promising results as an alternative treatment for fasciitis and is being explored for utilization in compartment syndrome, osteoarthritis, epicondylosis, myofascial pain syndrome, and other chronic pain syndromes [62,63]. 

Another possible indication is preoperative BoNT injection into the lateral abdominal muscle groups before the reconstruction of the abdominal wall. This adjunct therapy can induce temporary paralysis and elongation of the lateral abdominal muscles to create a tension-free closure and improved postoperative recovery course [64]. Motii et al. utilized BoNT preoperatively and achieved tension-free closure in complex hernia cases, without the need for extensive dissection [64]. A 2017 review examined the use of BoNT-A for abdominal wall reconstruction for incisional hernia. The study found that BonT-A given preoperatively can increase muscle length and facilitate primary fascial closure in 83.5% of patients without postoperative complications or adverse events [65]. 

Diastasis recti is another condition that may benefit from BoNT, which is characterized by the separation of the rectus abdominis muscles along the midline of the abdomen [66]. This condition is most commonly seen in postpartum women but can also occur in men and women with significant weight fluctuations or as a result of certain surgical procedures. Diastasis recti can lead to functional impairments, including reduced core strength and stability, as well as aesthetic concerns [66]. Several open or laparoscopic surgical procedures are available for diastasis recti management, with technique choice depending on the inter-rectus distance of the anterior abdominal wall laxity [67]. However, there are no distinct guidelines for treatment choice, although very large diastases can be treated with BoNT infiltration before surgery to enhance the laxity of the abdominal musculature [67]. However, BoNT can potentially be utilized in this condition to reduce or prevent the need for surgery. By injecting BoNT into the lateral abdominal muscles, it may be possible to reduce tension on the linea alba, allowing the separated rectus abdominis muscles to realign and close the gap [68]. This could be particularly beneficial when combined with physical therapy aimed at strengthening the core muscles. Ultrasound is another important component to assist in visualizing the extent of muscle separation and individual anatomical variations before BoNT treatment [64]. Although there are no current guidelines for this procedure, similar to the procedure for complex hernia surgeries, the authors suggest that 200 units of BoNT in 25 unit aliquots can be evenly injected into the bilateral rectus abdominis to facilitate medialization of the abdominal wall as done by Motii et al. [64] and discussed by Whitehead-Clarke and Windsor [69]. The dosage of BoNT in the abdominal wall has ranged from 100 to 500 units, but recent studies have opted for a reduced dosage of 200 units [69]. Injection intervals/follow-ups can be performed at one, three, and six months based on the typical duration of BoNT (three to four months). At follow-ups, evaluation of muscle tension, symptom improvement, and ultrasound measurements of diastasis width are important to assess for progression or reduction. If patients are symptomatic, standardized scales such as the visual analog scale can be used to track symptoms over time [69]. However, there remains the need for further studies and clinical trials to determine the efficacy, safety, and optimal protocols for BoNT use in these conditions. Additionally, an important consideration is the precision of administration to avoid unintentional paralysis of other structures.

Gastrointestinal (GI)

BoNT, due to its mechanism of action of acetylcholine release inhibition, provides an alternative therapeutic option for spastic disorders of the GI system. In the esophagus, BoNT has been utilized for achalasia, cricopharyngeal dysphagia, cricopharyngeal achalasia in children, diffuse esophageal spasm, non-cardiac chest pain, ineffective esophageal motility, and isolated lower esophageal sphincter (LES) dysfunction [70]. However, the results may vary depending on the specific esophageal disorder and the response is typically transient. For gastric applications, BoNT has been used for gastroparesis, obesity (transiently decreased gastric emptying), pyloric stenosis, pyloric obstruction, and facilitating gastric emptying after a surgical procedure. In the biliary system, BoNT can be used for sphincter of Oddi dysfunction, decreasing the incidence of postoperative pancreatic fistula, and acalculous biliary pain. Finally, for pelvic and anorectal conditions, BoNT can be utilized for pelvic floor dyssynergia, outlet obstruction/constipation, chronic idiopathic anal pain, anal fissures, internal anal sphincter dysfunction, Hirschsprung’s disease, and pain after hemorrhoidectomy [70]. 

Another possible indication is intrapyloric BoNT injection for feeding disorders and associated GI symptoms. Hirsch et al. found that 57/85 (67%) patients had partial or complete improvement in symptoms. Notably, of the patients who were receiving enteral tube feeding, there was an increased number of oral feeds after BoNT (p=0.004) and a decreased need for post-pyloric feeds (p=0.01) [71]. A meta-analysis of a small cohort of 20 children also found efficacy in providing transient relief (68% response, 95% CI: 59-78) of upper GI symptoms with and without gastroparesis (66%, 95% CI: 53-78) [72]. 

Due to its positive safety profile, it may be a valid option for patients requiring invasive procedures or for those who have a high risk of anesthesia or operative complications. Additionally, BoNT may be a worthwhile therapy to pursue if other medical interventions have failed and future investigations into GI disorder usage may find further indications for novel BoNT treatment. 

Neurological

Achalasia, a relatively uncommon disorder occurring in roughly 10 cases per 100,000 individuals, is a condition that disrupts people's ability to swallow comfortably [73]. Pathologically it is a progressive neurological degeneration of ganglion cells in the myenteric plexus of the esophageal wall. This causes improper relaxation of the lower esophageal sphincter and loss of peristalsis in the distal esophagus, often presenting as gastroesophageal reflux disease (GERD) or other related symptoms. BoNT has been utilized for achalasia and other spastic motor disorders via an endoscopic intrasphincteric route. Kumar et al. studied its effects on the LES, hiatal contraction, and gastroesophageal reflux. They found a significant decrease in esophagogastric junction pressure at end-expiration, tidal inspiration, forced inspiration, Muller’s maneuver, and straight leg raise [74]. However, after 6-12 months following the intervention, the LES and hiatal contraction pressure returned to pre-intervention levels. A safety analysis of 661 BoNT treatments in 386 patients found a total of 52 (7.9%) mild complications, consisting of chest pain and heartburn (n=29) or epigastric pain (n=5) [75]. However, younger patients appeared to have a higher rate of complications. Esophageal body injections, the number of injections per procedure, previous BoNT treatment, and greater concentration of BoNT injection were determined to not be risk factors for complications. 

Cerebral palsy (CP) is the most common motor development in children, occurring in about one in every 345 children in the United States [76]. It is caused by abnormalities of the developing fetal brain and affects motor dysfunction, muscle tone, and movement. An updated 2020 review found that many randomized, placebo-controlled trials have found BoNT to be efficacious in reducing spasticity and pain, improving range of motion (active/passive), helping progress functional and motor learning, and delaying orthopedic degradations [77]. Although not FDA-approved, BoNT is widely prescribed for the treatment of localized and troublesome limb spasticity from the age of two years [77]. However, a multidisciplinary team and a precise clinical evaluation using validated scales and instrumental analysis are essential in determining the probable outcome of BoNT treatment. 

In addition to these conditions, BoNT has been a safe and effective treatment for a variety of other hyperkinetic movement disorders and spastic conditions (excessive muscle activity/tone). More recent research has expanded on its role in the treatment of neurodegenerative diseases as well [78,79]. 

Psychological

The facial feedback hypothesis posits that feedback from facial expressions can influence subjective experiences of emotion. Psychiatric conditions also frequently are accompanied by facial expressions that reflect mood and personality disturbances. Thus, given this hypothesis, disruption of this feedback loop using BoNT can provide a therapeutic benefit [80]. 

A 2024 meta-analysis of 21 studies with 471 patients focused on trials that evaluated BoNT’s effects on major depressive disorder, borderline personality disorder, social anxiety, and bipolar disorder [81]. The most common area for injection was the glabellar area which is responsible for the expression of frowning. Results across these studies demonstrated significant symptom reductions for each psychiatric condition utilizing symptom rating instruments that are aligned with their respective clinical focus. However, despite these promising findings, there was a paucity of biomarker-related assessments to assess for neurobiological changes after treatment. A handful of studies have found that BoNT treatment downregulates the amygdala and other pathways involved in the emotion-to-motor transformation loop. Currently, BoNT injections into the glabella do represent a promising novel method of treating psychiatric conditions, and further research into its role in disorders with an affectivity component is still underway [81].

Cardiovascular

In a randomized double-blind study conducted on patients with a history of paroxysmal atrial fibrillation and who had indications for coronary artery bypass graft surgery, BoNT was able to substantially suppress atrial tachyarrhythmia early on as well at the one-year follow-up compared to the placebo group (p=0.002) [82]. At a three-year follow-up of a study, the incidence of atrial tachyarrhythmia was 23.3% in the BoNT group and 50% in the placebo group (p=0.02), with significantly lower atrial fibrillation burden, noted at one year, two years, and three years [83]. However, a 2024 meta-analysis of three randomized controlled trials found no difference between BoNT and placebo for preventing postoperative atrial fibrillation. Thus, more studies are required to elucidate BoNT’s role in managing cardiovascular conditions. Although BoNT treatment for these conditions has yet to be FDA-approved, preliminary results are promising and there has been interest in exploring BoNT’s usage in cardiovascular conditions as well such as ischemia-reperfusion injuries, hypertension, atrial fibrillation, and heart failure [84]. 

The non-FDA-approved uses of BoNT have been summarized in Table 2. 

**Table 2:** Non-FDA approved uses

Systems	Conditions
Musculoskeletal [40-69]	Bruxism
	Temporomandibular joint disorder
	Wound healing/scarring
	Cleft lip repair
	Reduced oral aperture
	Raynaud’s phenomenon
	Plantar Fasciitis
	Compartment syndrome
	Osteoarthritis
	Epicondylosis
	Myofascial pain syndrome
	Myofascial pelvic pain syndrome
	Piriformis syndrome
	Fibromylagia
	Cervicogenic headache
	Chronic musculoskeletal pain
	Lateral abdominal wall relaxation before hernia repair
	Diastasis recti
Gastrointestinal [70-72]	Achalasia
	Cricopharyngeal dysphagia
	Cricopharyngeal achasia
	Diffuse esophageal spasm
	Non-cardiac chest pain
	Ineffective esophageal motility
	Isolated lower esophageal sphincter dysfunction
	Gastroparesis
	Obesity
	Pyloric stenosis
	Pyloric obstruction
	Gastric emptying after a surgical procedure
	Sphincter of Oddi dysfunction
	Decreasing postoperative pancreatic fistula
	Acalculous biliary pain
	Pelvic floor dyssnergia
	Pelvic outlet obstruction/constipation
	Chronic idiopathic anal pain
	Anal fissures
	Internal anal sphincter dysfunction
	Hirschsprung’s disease
	Pain after hemorrhoidectomy
Neurological [73-79]	Achalasia
	Lower esophageal sphincter dysfunction
	Hiatal contraction
	Gastroesophageal reflux
	Cerebral palsy
	Hyperkinetic movement disorders
	Spastic conditions
	Dystonia
	Tics
	Tremors
	Myoclonus
	Restless legs syndrome
	Tardive dyskinesia
Psychological [80,81]	Major depressive disorder
	Borderline personality disorder
	Social anxiety disorder
	Bipolar disorder
Cardiovascular [83,84]	Paraoxysmal atrial fibrillation
	Atrial tachyarrhythmia

## Conclusions

BoNT has established indications across various medical specialties. Looking beyond its current applications, BoNT holds promise in various medical fields but is not FDA-approved in these conditions. However, with these widespread indications, there is significant heterogeneity observed in the current literature comprising sample selection, measurement outcomes, measurement procedure, and injection protocol, which prevents the definitive conclusion of the efficacy of BoNT. Thus, the design and conduction of well-designed randomized controlled trials are essential in establishing the efficacy and safety of BoNT treatment which can help accelerate regulatory approval for new indications.
